# Necrotizing Fasciitis With Myonecrosis in a Diabetic Patient: Highlighting the Role of Early Detection and Management

**DOI:** 10.7759/cureus.81720

**Published:** 2025-04-04

**Authors:** Bano Alsaleh, Ahmed Alanzi, Dawood Alatefi, Mohammed Alsaleh, Ahmed Alsaleh, Fouad Aladel

**Affiliations:** 1 Radiology, King Hamad University Hospital, Muharraq, BHR; 2 Anesthesia and Critical Care, King Hamad University Hospital, Muharraq, BHR; 3 Faculty of Medicine, University of Jordan Hashemite, Amman, JOR; 4 Pediatric Medicine, Mohammed Bin Khalifa Bin Salman Al Khalifa Specialist Cardiac Centre, Awali, BHR; 5 Faculty of Medicine, Royal College of Surgeons in Ireland, Muharraq, BHR; 6 Radiology, King Fahad Specialist Hospital Dammam, Dammam, SAU

**Keywords:** diabetes, early detection, imaging, myonecrosis, necrotizing fasciitis

## Abstract

Necrotizing fasciitis (NF) is a life-threatening soft tissue infection that progresses rapidly and can lead to systemic complications. Myonecrosis, a severe complication of NF, involves muscle tissue death and often requires aggressive treatment. A 35-year-old female with diabetes mellitus, dyslipidemia, obesity, and a history of right breast cancer presented with acute, progressive right thigh pain, fever, and vomiting. Physical examination revealed local swelling, tenderness, warmth, and systemic signs of infection. Laboratory tests showed leukocytosis, elevated C-reactive protein, renal impairment, and hyponatremia. Contrast-enhanced MRI of the right thigh raised suspicion of NF with focal myonecrosis in the vastus lateralis and intermedius muscles. Surgical exploration and histopathology confirmed NF and myonecrosis. Debridement and broad-spectrum antibiotics, including vancomycin, meropenem, and clindamycin, were started. A second debridement and follow-up MRI showed improvement, with the patient recovering well and being discharged without complications. Early imaging, aggressive surgical intervention, and appropriate antibiotic therapy are critical in managing NF and myonecrosis, particularly in high-risk patients.

## Introduction

Necrotizing fasciitis (NF) is a rapidly progressing soft tissue infection that can lead to widespread tissue necrosis, systemic shock, and high mortality if not diagnosed and treated promptly. The infection affects the fascial planes and can involve the skin, subcutaneous tissue, and muscles [1]. It is classified into four types: type 1 (polymicrobial), type 2 (monomicrobial, caused by group A β-hemolytic streptococci or *Staphylococcus aureus*), type 3 (monomicrobial, caused by Gram-negative bacilli such as *Escherichia coli*), and type 4 (fungal infection) [2]. Myonecrosis, or the death of muscle tissue, is a rare but severe complication of NF. Risk factors include diabetes, obesity, immunocompromised states, and prior treatments for malignancy. This case presents a patient with NF complicated by myonecrosis, emphasizing the importance of early diagnosis, imaging, and surgical intervention.

## Case presentation

A 35-year-old female with a history of type 2 diabetes mellitus, dyslipidemia, obesity (class 1), and previous right breast cancer (metaplastic carcinoma with squamous differentiation, stage pTx N1a 2/39 M0, Ki-67 50%), who had undergone wide local excision and axillary clearance with subsequent chemotherapy and radiotherapy in 2015, later developed compensated chemo-induced cardiomyopathy.

The patient presented to the emergency department with a three-day history of progressive right thigh pain, fever (38-39°C), vomiting, and decreased oral intake. She also reported decreased urine output but denied urinary symptoms. The pain was continuous, nonradiating, and not associated with physical activity. She denied any trauma or previous similar pain episodes. On examination, the right thigh showed swelling, tenderness, and warmth, but no crepitation or skin changes. Distal pulses were intact, and neurological examination could not be properly assessed due to pain.

The patient’s laboratory workup revealed an elevation in the white blood cell count with a left shift (increased neutrophils), indicating an ongoing infection, along with a significantly elevated C-reactive protein (CRP) level, a marker of inflammation. Additionally, the patient’s renal function was impaired, as evidenced by elevated blood urea nitrogen and creatinine levels. Hemoglobin was decreased, and the estimated glomerular filtration rate was reduced, suggesting possible renal compromise (Table 1).

**Table 1:** Laboratory workup of the patient

Investigation	Patient value	Reference value
Complete blood count		
Hemoglobin	6.2 gm/dL	12.5-15.0 gm/dL
White blood cells	12 × 10⁹/L	4.5-11.0 × 10⁹/L
Neutrophils	9.2 × 10⁹/L	2.0-7.5 × 10⁹/L
Lymphocytes	0.98 × 10⁹/L	1.0-3.0 × 10⁹/L
C-reactive protein	353 mg/L	<5 mg/L
Renal function test		
Blood urea nitrogen	9.4 mmol/L	2.5-7.5 mmol/L
Creatinine	192 µmol/L	45-90 µmol/L
Glomerular filtration rate	27 mL/min/1.73 m²	>90 mL/min/1.73 m²
Sodium	122 mmol/L	135-145 mmol/L
Potassium	3.8 mmol/L	3.5-5.1 mmol/L

**Figure 1:**
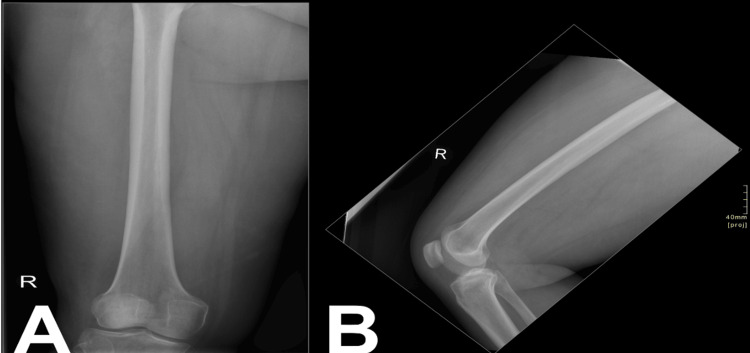
Anteroposterior (A) and lateral (B) projections of the right femur and right knee joint show no fracture lines, preserved joint spaces, no joint effusion, and no aggressive osseous lesions

**Figure 2:**
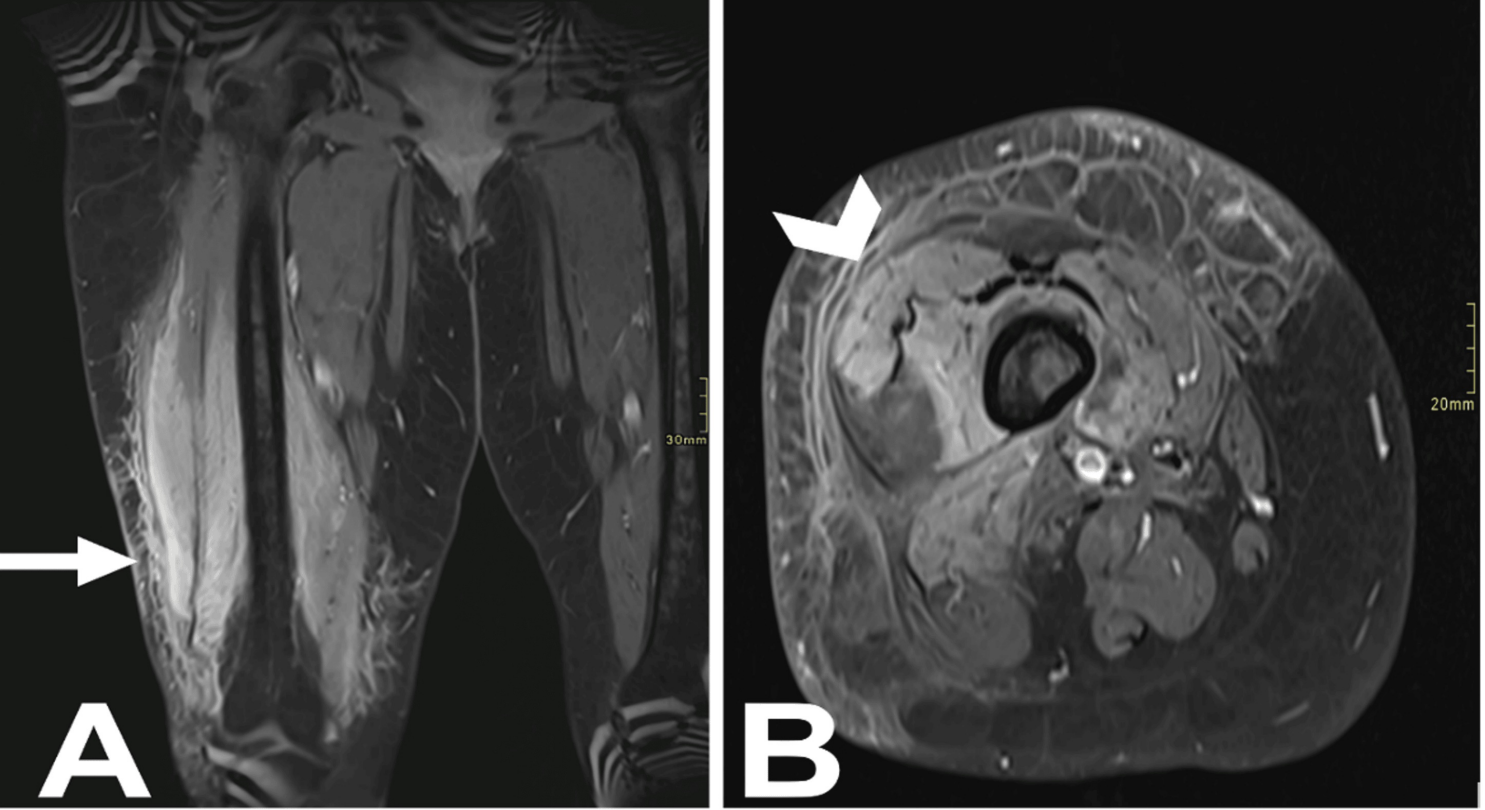
Two selected MRI images of the right thigh, obtained post-IV gadolinium injection in coronal (A) and axial (B) cuts, show marked edema and enhancement of the subcutaneous tissue on the anterior and lateral aspects of the right thigh

**Figure 3:**
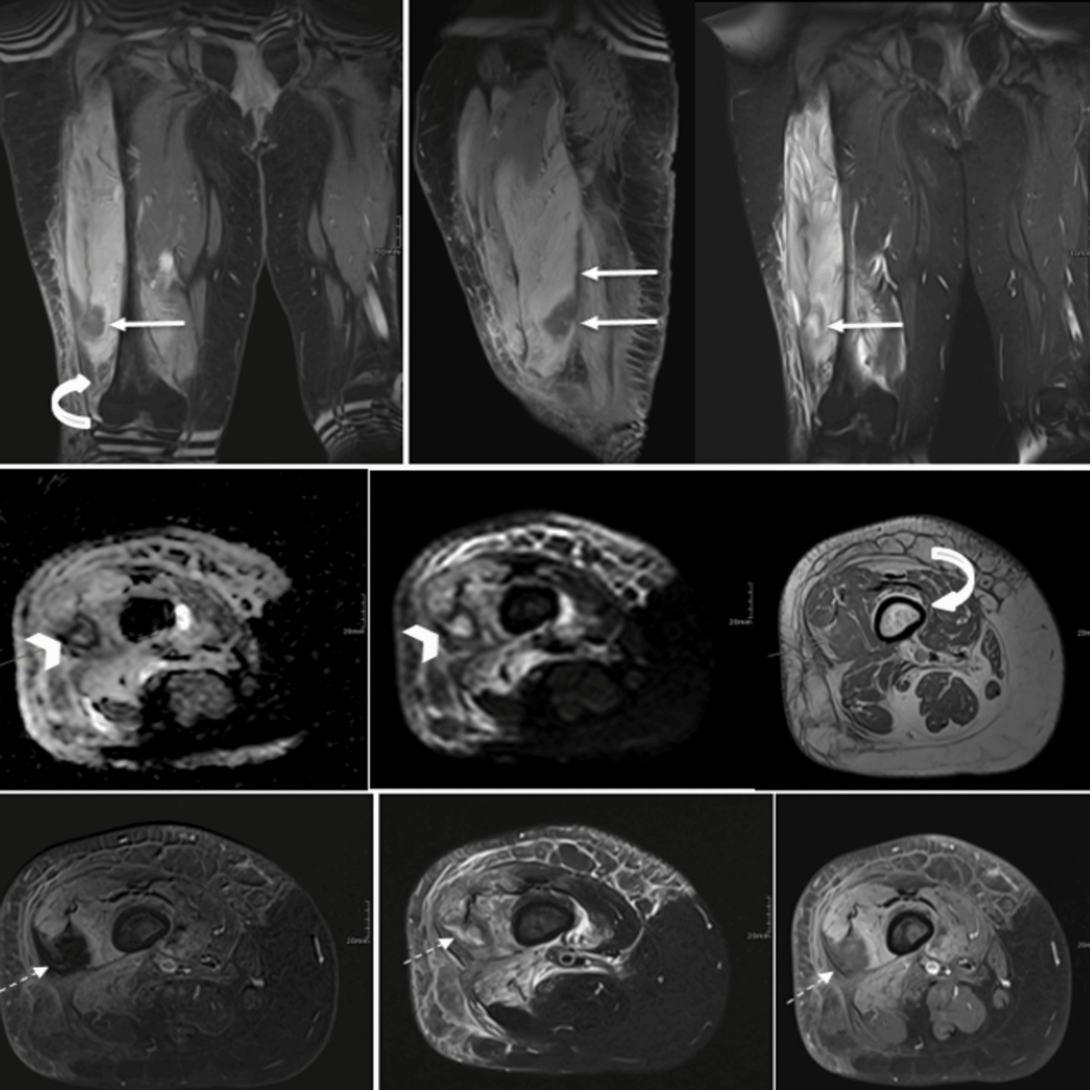
Multisequential and multiplanar MRI of the right thigh, obtained pre- and post-IV gadolinium administration, shows the distal portion of the vastus lateralis/intermedius demonstrating a focal area of distorted architecture (dotted arrow) with diffusion restriction (arrowhead) that lacks enhancement (arrow), representing myonecrosis. This area measures 2.4 × 2.7 × 10.6 cm in AP × TR × CC dimensions. No definite gas foci are detected. Mild edema and enhancement are noted in the biceps femoris muscle. The bilateral femurs show background heterogeneous marrow signal changes and enhancement (curved arrow), likely related to red marrow activation secondary to underlying conditions. No bone marrow-replacing lesion is present to suggest osteomyelitis

Radiographic imaging of the right thigh did not reveal any fractures (Figure 1). A contrast-enhanced MRI of the thigh showed significant soft tissue edema and enhancement, primarily involving the anterior and lateral compartments, including the underlying muscles. Notably, a focal area of distorted tissue architecture was observed in the distal vastus lateralis/intermedius, which demonstrated diffusion restriction and lacked enhancement, raising suspicion for NF with myonecrosis. No gas foci were observed, and there was no evidence of bone marrow involvement to suggest osteomyelitis (Figure 2, Figure 3). These MRI findings were consistent with a diagnosis of cellulitis, fasciitis, and myositis, along with myonecrosis in the vastus lateralis/intermedius, which prompted the need for urgent surgical intervention.

The patient underwent surgical exploration and debridement of the right thigh. Histopathological examination confirmed acute NF and myonecrosis. Broad-spectrum antibiotics were initiated, including a STAT dose of vancomycin, along with meropenem and clindamycin. The patient was also transfused with one unit of packed red blood cells. Two days later, a second debridement was performed, and follow-up MRI revealed a reduction in the nonenhancing areas, as well as minimal fluid accumulation, indicating clinical improvement.

The patient recovered without complications and was discharged, with no evidence of recurrence at the follow-up visit.

## Discussion

NF is a rare disease that affects men approximately ten times more often than women [3,4]. The prevalence of NF is about 0.3-15 cases per 100,000 people [5]. However, the actual incidence of NF may be higher, as it is often difficult to diagnose and lacks specific symptoms, making it easy to miss. When diagnosis is delayed, the disease can spread rapidly, leading to more severe tissue damage and a worse prognosis for recovery [6,7]. Common signs include localized tenderness or itching, skin redness, and fever. Initially, the skin around the affected area may appear normal, but within a few days, it becomes warm, red, and increasingly tender. In the present case, the patient had swelling in the right thigh, with tenderness upon palpation and warmth. Tissue necrosis typically begins to appear three to five days after the onset of symptoms [8].

NF generally has a high mortality rate, reported between 20% and 35% in various studies [6,9]. The infection can result from even a minor wound or scratch. NF leads to extensive, rapidly advancing necrosis involving the skin, subcutaneous tissue, and fascia. The necrosis is caused by microemboli within the vasculature of the affected tissues. NF is typically observed in the lower extremities and anogenital region, with less frequent occurrences in the soft tissues of the upper extremities or the head and neck region [9]. In the present case, the patient’s underlying risk factors, such as diabetes, obesity, and a history of chemotherapy-induced cardiomyopathy following breast cancer treatment, likely contributed to her vulnerability to NF. Diabetes is a well-documented risk factor for NF due to its association with vascular disease, impaired immune response, and reduced wound healing capacity [10]. Other significant risk factors for NF include obesity and smoking. Obesity complicates NF further due to decreased vascular perfusion, which can inhibit antibiotic delivery to infected tissues, increasing susceptibility to rapid bacterial proliferation [11]. A retrospective study of 127 patients with NF investigated differences between diabetic and nondiabetic patients [12]. The findings indicated that 61.4% of all diagnosed NF patients were diabetic. Furthermore, diabetic patients had more polymicrobial infections, end-stage renal disease, and comorbidities. Diabetic patients were also more likely to experience misdiagnosis and higher rates of amputation [12].

In our patient, MRI played a central role in diagnosing NF and assessing the extent of myonecrosis. MRI has been shown to be one of the most sensitive imaging modalities for soft tissue infections due to its ability to identify edema, fascial thickening, and deep tissue involvement [13]. The extensive edema and enhancement of the subcutaneous tissues, fascial planes, and muscles in our patient were consistent with early-stage NF, as described in multiple studies [14,15]. Unlike CT scans or X-rays, MRI is more effective in distinguishing between cellulitis, fasciitis, and myositis, which are often difficult to differentiate clinically due to overlapping symptoms [16]. CT imaging may show gas formation in the later stages of NF, but gas is often absent in early presentations or nongas-forming infections, as seen in this case [17]. Gas formation is caused by facultative anaerobes.

Laboratory findings, including elevated CRP, leukocytosis, and renal impairment, further supported the diagnosis. Elevated CRP levels have been shown to correlate with the severity of inflammation and infection in NF patients [18,19]. Studies suggest that a CRP level above 150 mg/L strongly indicates necrotizing infections, with higher CRP levels often associated with increased tissue necrosis and mortality. A systematic review by Tarján et al. reported that CRP and procalcitonin are good predictors of infection in NF [20]. Treatment of NF is multifaceted, combining surgical debridement, broad-spectrum antibiotics, and supportive care. Immediate and aggressive surgical debridement is essential to remove necrotic tissue and halt the infection's spread. In this case, the patient underwent two debridements, which were critical in managing the infection and preventing systemic involvement. The use of broad-spectrum antibiotics, such as vancomycin, meropenem, and clindamycin, covers a wide range of possible pathogens. Vancomycin and meropenem target methicillin-resistant *S. aureus* and resistant Gram-negative bacteria, while clindamycin, by inhibiting toxin production, is beneficial in NF, where toxin-producing bacteria can exacerbate tissue destruction [21,22].

## Conclusions

NF with myonecrosis is a life-threatening condition that requires prompt diagnosis and aggressive treatment. MRI plays a crucial role in early detection, and timely surgical debridement, along with broad-spectrum antibiotics, significantly improves patient prognosis. High-risk patients, such as those with diabetes and obesity, should be closely monitored for signs of infection to ensure early intervention. This case report highlights the critical role of early imaging, prompt surgical intervention, and broad-spectrum antibiotic therapy in managing NF, particularly in high-risk patients with diabetes and prior immunocompromise. Furthermore, interdisciplinary management should be considered in some cases to reduce the risk of complications.

## References

[1] LaChance A, Kroshinksy D (2019). Necrotizing fasciitis, necrotizing cellulitis, and myonecrosis. Fitzpatrick's Dermatology, 9th Edition.

[2] Sarkardeh M, Ahmadabadi A, Sadrzadeh Z, Koushki J, Esmaeili A, Davoodi S, Izanlu M (2022). Extensive monomicrobial necrotizing fasciitis and myonecrosis of left hemi trunk in a healthy 41-year-old man with COVID-19 infection. Iran J Microbiol.

[3] Wang JM, Lim HK (2014). Necrotizing fasciitis: eight-year experience and literature review. Braz J Infect Dis.

[4] Chen LL, Fasolka B, Treacy C (2020). Necrotizing fasciitis: a comprehensive review. Nursing.

[5] Stevens DL, Bryant AE (2017). Necrotizing soft-tissue infections. N Engl J Med.

[6] Arif N, Yousfi S, Vinnard C (2016). Deaths from necrotizing fasciitis in the United States, 2003-2013. Epidemiol Infect.

[7] Kiat HJ, En Natalie YH, Fatimah L (2017). Necrotizing fasciitis: how reliable are the cutaneous signs?. J Emerg Trauma Shock.

[8] Salati SA (2022). Necrotizing fasciitis - a review. Pol Przegl Chir.

[9] Kapi E, Dogan ZDA, Seyhan T (2018). Unusual cases of necrotizing fasciitis: a clinical experience from Turkey. Eur J Plast Surg.

[10] Hodgins N, Damkat-Thomas L, Shamsian N, Yew P, Lewis H, Khan K (2015). Analysis of the increasing prevalence of necrotising fasciitis referrals to a regional plastic surgery unit: a retrospective case series. J Plast Reconstr Aesthet Surg.

[11] Villela NR, Kramer-Aguiar LG, Bottino DA, Wiernsperger N, Bouskela E (2009). Metabolic disturbances linked to obesity: the role of impaired tissue perfusion. Arq Bras Endocrinol Metabol.

[12] Tan JH, Koh BT, Hong CC (2016). A comparison of necrotising fasciitis in diabetics and non-diabetics: a review of 127 patients. Bone Joint J.

[13] Spinnato P, Patel DB, Di Carlo M, Bartoloni A, Cevolani L, Matcuk GR, Crombé A (2022). Imaging of musculoskeletal soft-tissue infections in clinical practice: a comprehensive updated review. Microorganisms.

[14] Wei XK, Huo JY, Yang Q, Li J (2024). Early diagnosis of necrotizing fasciitis: Imaging techniques and their combined application. Int Wound J.

[15] Kochkine S, Payne DL, Chung K, Chen D, Bernstein MP, Baxter AB, McMenamy JM (2024). Imaging of necrotizing fasciitis. Clin Imaging.

[16] Matcuk GR Jr, Skalski MR, Patel DB (2024). Lower extremity infections: essential anatomy and multimodality imaging findings. Skeletal Radiol.

[17] Carbonetti F, Cremona A, Carusi V (2016). The role of contrast enhanced computed tomography in the diagnosis of necrotizing fasciitis and comparison with the laboratory risk indicator for necrotizing fasciitis (LRINEC). Radiol Med.

[18] Borschitz T, Schlicht S, Siegel E, Hanke E, von Stebut E (2015). Improvement of a clinical score for necrotizing fasciitis: 'pain out of proportion' and high CRP levels aid the diagnosis. PLoS ONE.

[19] Zil-E-Ali A, Fayyaz M, Fatima A, Ahmed Z (2018). Diagnosing necrotizing fasciitis using procalcitonin and a laboratory risk indicator: brief overview. Cureus.

[20] Tarján D, Szalai E, Lipp M (2024). Persistently high procalcitonin and C-reactive protein are good predictors of infection in acute necrotizing pancreatitis: a systematic review and meta-analysis. Int J Mol Sci.

[21] Stevens DL, Bisno AL, Chambers HF (2005). Practice guidelines for the diagnosis and management of skin and soft-tissue infections. Clin Infect Dis.

[22] Legbo JN, Shehu BB (2005). Necrotizing fasciitis: a comparative analysis of 56 cases. J Natl Med Assoc.

